# Supplementary material for: Boron Triangular Kagome Lattice with Half-Metallic Ferromagnetism
Source: Sci Rep. 2017 Aug 4;7:7279. doi: 10.1038/s41598-017-07518-9 (PMC5544701; doi:10.1038/s41598-017-07518-9)
Supplement: Supplementary file 1 — Supplementary information [file 41598_2017_7518_MOESM1_ESM.pdf]

Supplementary Information for: Boron Triangular Kagome Lattice with Half-Metallic Ferromagnetism

Sunghyun Kim,*,† Woo Hyun Han,† In-Ho Lee,‡ and K. J. Chang*,†

†*Department of Physics, Korea Advanced Institute of Science and Technology, Daejeon 34141, Korea*

‡ *Korea Research Institute of Standards and Science, Daejeon 34113, Korea*

E-mail: kimsunghyun@kaist.ac.kr; kjchang@kaist.ac.kr

Phone: +82-42-350-2571. Fax: +82-42-350-2510

Structure stability

The energy of B_9-tKL is higher by 90 meV/B than that of the B α-sheet, whereas it is comparable to the value 70 meV/B for the borophene structure. We examined the stability of B_9-tKL and B_9-KL (under 18% strain) by calculating the full phonon spectra and find no imaginary phonon modes, indicating that both the Kagome lattices are dynamically stable (see Fig. 4 in the main text). In addition, we performed first-principles molecular dynamics simulations for 100 ps to examine the thermal stability of B_9-tKL and B_9-KL. We find that B_9-tKL and B_9-KL are stable at temperatures up to 1000 K and 400 K, respectively (Fig. S1). Due to the strain effect, B_9-KL is higher in energy by about 470 meV/B than B_9-tKL. Thus, the crystal structure of B_9-KL becomes unstable as temperature increases above 400 K.

Method

To investigate the orbital characteristics of the nearly flat band in B_9-KL, we built a simplified tight-binding Hamiltonian by considering only the nearest-neighbor interactions. Due to the small difference between t_1 and t_2, we set $t = t_1 = t_2$ to further simplify the Hamiltonian. Then, the tight-binding Hamiltonian is given by

$$\mathcal{H} = t \sum_{\langle i_E, j_E \rangle} c^{\dagger}_{i_E} c_{j_E} + t \sum_{\langle i_E, j_C \rangle} c^{\dagger}_{i_E} c_{j_E} + \text{H.C.}, \tag{S1}$$

where $\langle i_E, j_E \rangle$ denotes nearest-neighbor pairs between the edge B atoms, $\langle i_E, j_C \rangle$ stands for nearest-neighbor pairs between the edge and corner B atoms, and $c^{\dagger}_i$ (c_i) is the creation (annihilation) operator on the site i (see numbers in Fig. S3). The eigenvalue of the flat band is t and its eigenstate $|\epsilon(\mathbf{k}) = t\rangle$ is expressed as

$$\begin{aligned} |\epsilon(\mathbf{k}) = t\rangle = &-\frac{1}{2}(e^{i\mathbf{k}\cdot\mathbf{a}_2} - 1)c^{\dagger}_1(\mathbf{k}) + \frac{1}{2}(e^{i\mathbf{k}\cdot\mathbf{a}_1} - 1)c^{\dagger}_2(\mathbf{k}) - \frac{1}{2}(e^{i\mathbf{k}\cdot\mathbf{a}_1} - e^{i\mathbf{k}\cdot\mathbf{a}_2})c^{\dagger}_3(\mathbf{k}) \\ &-\frac{1}{2}e^{i\mathbf{k}\cdot\mathbf{a}_1}(e^{i\mathbf{k}\cdot\mathbf{a}_2} - 1)c^{\dagger}_4(\mathbf{k}) + \frac{1}{2}e^{i\mathbf{k}\cdot\mathbf{a}_2}(e^{i\mathbf{k}\cdot\mathbf{a}_1} - 1)c^{\dagger}_5(\mathbf{k}) - \frac{1}{2}(e^{i\mathbf{k}\cdot\mathbf{a}_1} - e^{i\mathbf{k}\cdot\mathbf{a}_2})c^{\dagger}_6(\mathbf{k}) \\ &+(e^{i\mathbf{k}\cdot\mathbf{a}_2} - 1)c^{\dagger}_7(\mathbf{k}) - (e^{i\mathbf{k}\cdot\mathbf{a}_1} - 1)c^{\dagger}_8(\mathbf{k}) + (e^{i\mathbf{k}\cdot\mathbf{a}_1} - e^{i\mathbf{k}\cdot\mathbf{a}_2})c^{\dagger}_9(\mathbf{k}), \end{aligned} \tag{S2}$$

where $\mathbf{a}_1$ and $\mathbf{a}_2$ are the lattice vectors in B_9-KL. Since the eigenvalues of the eigenstates $|\epsilon(\mathbf{k}) = t\rangle$ are independent of the $\mathbf{k}$ point, any linear combination of them is also the eigenstate. Once the localized wave function is built around a single hexagonal plaquette [Fig. S3], it is clear that hoppings from the orbitals with the same magnitudes but opposite phases exactly cancel each other. As a consequence, the wave function localized around a hexagonal plaquette is prohibited from hopping to its adjacent plaquette.

Binding energy

We examined the possibility of Mg deintercalation from the free-standing Mg_1B_9 sheet by calculating the Mg binding energy defined as,

$$E_b = \mu_{\mathrm{Mg,bulk}} - [E_{\mathrm{tot}}(\mathrm{Mg_1B_9}) - E_{\mathrm{tot}}(\mathrm{B_9}\text{-}t\mathrm{KL})], \tag{S3}$$

where $E_{\mathrm{tot}}(\mathrm{Mg_1B_9})$ and $E_{\mathrm{tot}}(\mathrm{B_9}\text{-}t\mathrm{KL})$ are the total energies of the free-standing Mg_1B_9 and B_9-tKL sheets, respectively. Here $\mu_{\mathrm{Mg,bulk}}$ denotes the Mg chemical potential which is obtained from the total energy of Mg in bulk metal. The Mg binding energy is estimated to be about 0.7 eV/Mg, and this value is much smaller than those of other cathode materials used for Mg batteries, including layered magnesium borides.[1–3] In previous studies,[1,2] the Mg ions were shown to be easily dissolved in conventional electrolytes. Similarly, the Mg ions can be deintercalated from the exfoliated Mg_1B_9 sheet in electrolytes due to their small Mg binding energy. We also considered other chemical potentials of Mg on the Ag(111) surface and in a vacuum, which are denoted as $\mu_{\mathrm{Mg,Ag(111)}}$ and $\mu_{\mathrm{Mg,vacuum}}$, respectively. Here $\mu_{\mathrm{Mg,vacuum}}$ just corresponds to the total energy of a single Mg atom in vacuum, and $\mu_{\mathrm{Mg,Ag(111)}}$ is the energy difference between a Mg monolayer on the Ag substrate and the bare Ag surface. The Mg binding energies are calculated to be 0.14 and 2.21 eV/Mg for $\mu_{\mathrm{Mg,Ag(111)}}$ and $\mu_{\mathrm{Mg,vacuum}}$, respectively [Table S1]. On the other hand, in the case of the Mg_1B_9 allotrope on the Ag surface, the Mg binding energies are enhanced by about 1.35 eV/Mg [Table S1], indicating that it is easier to extract the Mg ions from the free-standing Mg_1B_9 sheet.

Table S1: The Mg binding energies (in units of eV/Mg) of the free-standing Mg_1B_9 sheet and the Mg_1B_9 sheet on the Ag(111) surface are compared for various Mg chemical potentials.

	$\mu_{\mathrm{Mg,bulk}}$	$\mu_{\mathrm{Mg,Ag(111)}}$	$\mu_{\mathrm{Mg,vacuum}}$
Free-standing Mg_1B_9	0.70	0.14	2.21
Mg_1B_9 on Ag(111)	2.04	1.49	3.55

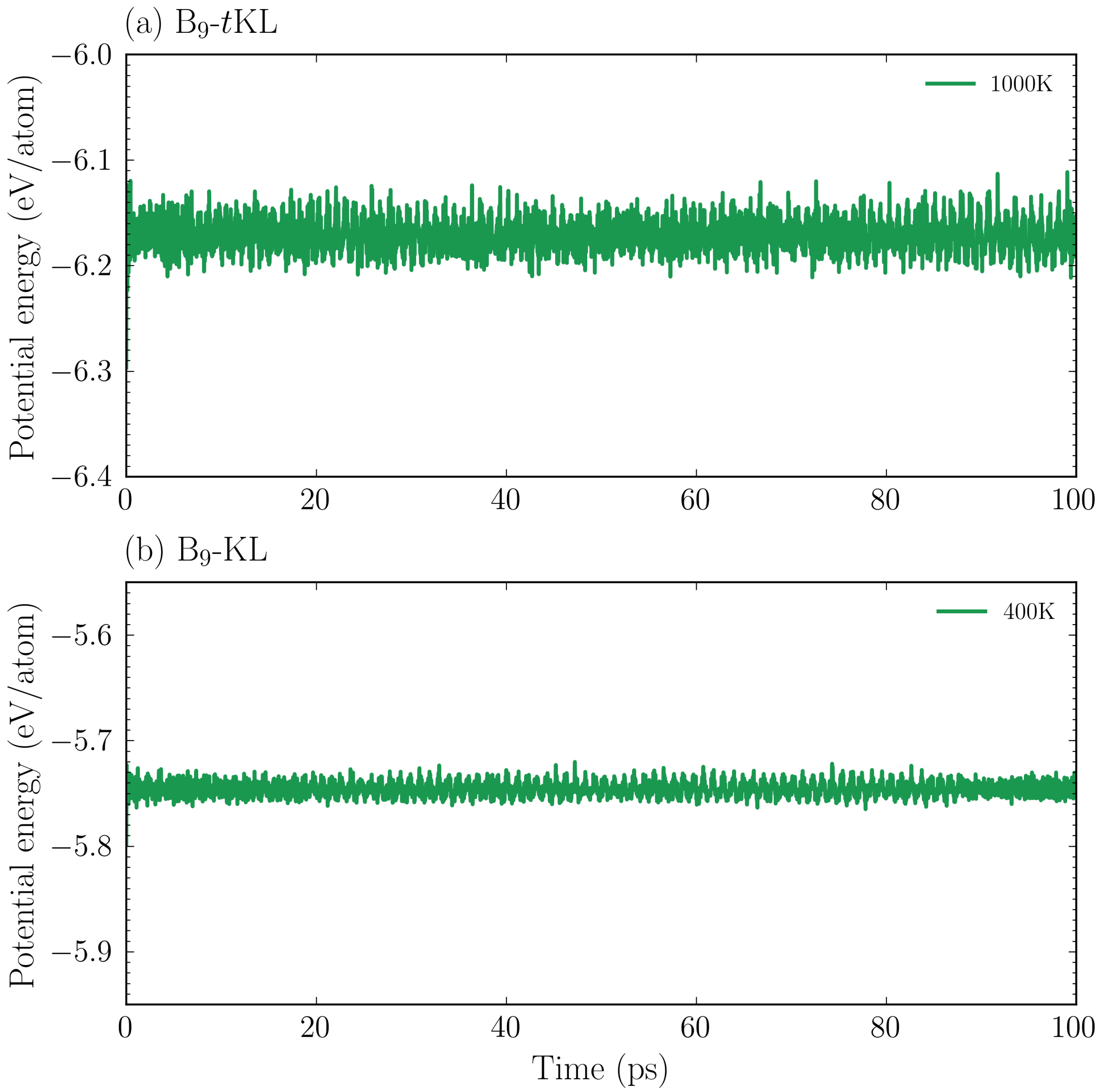

Figure S1: Potential energy fluctuations during first-principles molecular dynamics simulations at 1000 K for (a) B_9-tKL (without strain) and 400 K for (b) B_9-KL (under 18% strain).

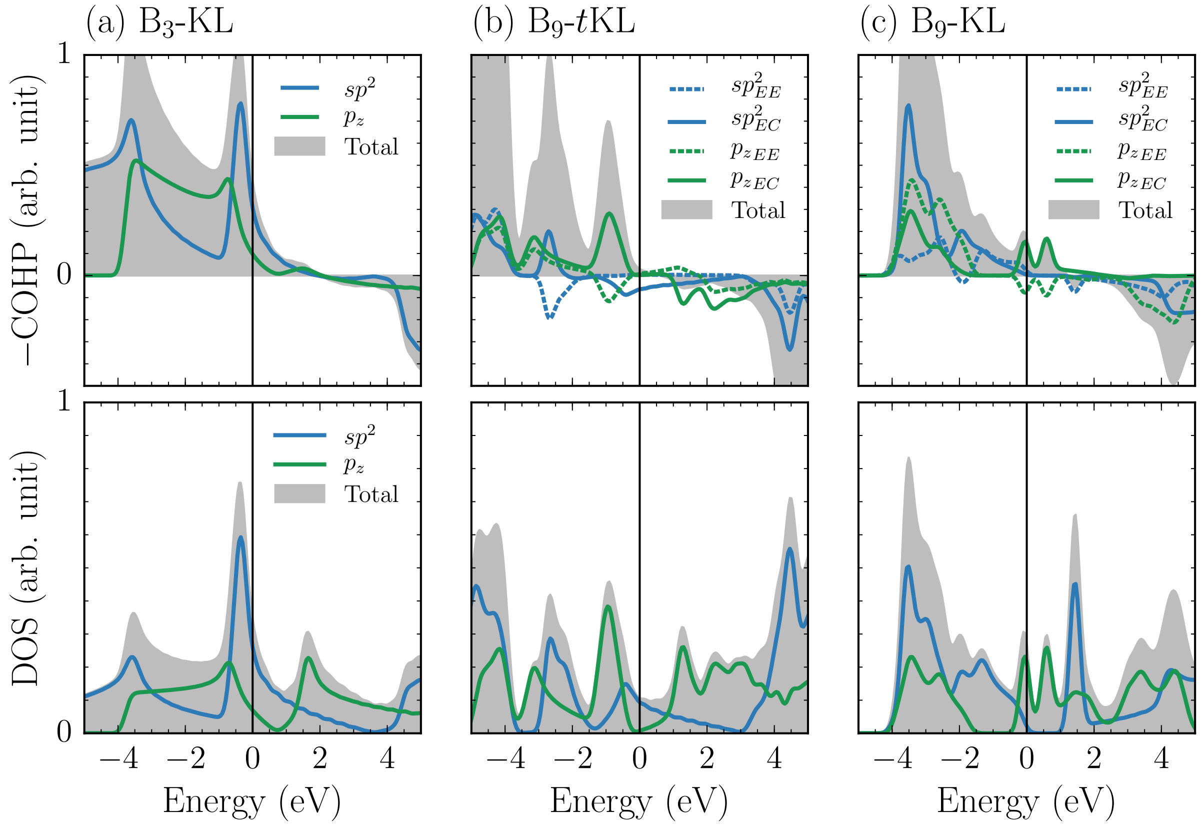

Figure S2: The negative of crystal orbital Hamilton population (−COHP)[4–6] and the density of states are plotted as a function of energy for (a) B_3-KL, (b) B_9-tKL, and (c) B_9-KL. The positive and negative values of −COHP indicate the bonding and antibonding characters in the density of states, respectively. Blue and green curves represent the densities of states projected onto the sp^2 and p_z orbitals of the B atoms, respectively. In (b) and (c), the subscripts E and C in the sp^2 and p_z hybridized orbitals denote the edge and corner B atoms in the large triangles of B_9-tKL and B_9-KL, respectively (see Fig. 3 in the main text).

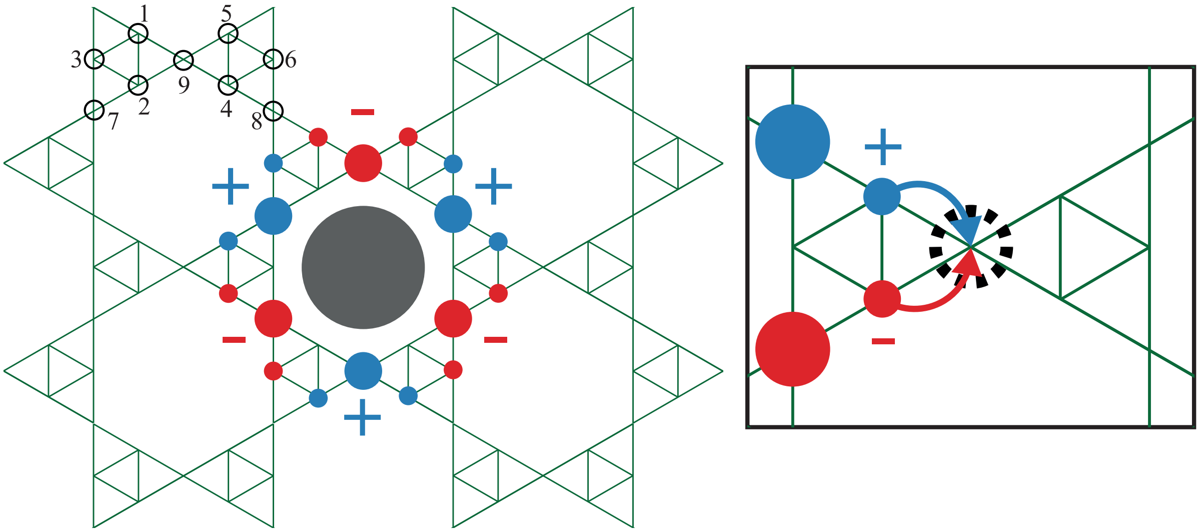

Figure S3: The wave function is drawn for the flat band near the Fermi level for up-spin electrons in B_9-KL, and it exhibits the localized nature around a hexagonal hole (shaded circle). Numbers denote the B sites in two corner-sharing triangles, and arrows indicate the hopping parameters in the effective tight-binding model. The size of blue (red) circles represents the magnitude of orbital weights with the positive (negative) phase. The inset shows a schematic illustration of the destructive interference of orbital hoppings to the adjacent hexagonal hole, indicating that the localized wave function is prohibited from propagating.

References

(1) Zhao, Y.; Ban, C.; Xu, Q.; Wei, S.-H.; Dillon, A. C. *Phys. Rev. B* **2011**, *83*, 035406.

(2) Saha, P.; Datta, M. K.; Velikokhatnyi, O. I.; Manivannan, A.; Alman, D.; Kumta, P. N. *Prog. Mater. Sci.* **2014**, *66*, 1–86.

(3) Zhang, R.; Ling, C. *MRS Energy Sustain.* **2016**, *3*, E1.

(4) Dronskowski, R.; Bloechl, P. E. *J. Phys. Chem.* **1993**, *97*, 8617–8624.

(5) Deringer, V. L.; Tchougreeff, A. L.; Dronskowski, R. *J. Phys. Chem. A* **2011**, *115*, 5461–5466.

(6) Maintz, S.; Deringer, V. L.; Tchougréeff, A. L.; Dronskowski, R. *J. Comput. Chem.* **2013**, *34*, 2557–2567.
